# The radiographic assessment of lung edema score of lung edema severity correlates with inflammatory parameters in patients with coronavirus disease 2019—Potential new admission biomarkers to predict coronavirus disease 2019 worsening

**DOI:** 10.3389/fmed.2022.871714

**Published:** 2022-08-11

**Authors:** Patrice Marques, Lucia Fernandez-Presa, Aitor Carretero, Maria-Carmen Gómez-Cabrera, José Viña, Jaime Signes-Costa, Maria-Jesus Sanz

**Affiliations:** ^1^Department of Pharmacology, Faculty of Medicine and Odontology, University of Valencia, Valencia, Spain; ^2^Institute of Health Research INCLIVA, University Clinic Hospital of Valencia, Valencia, Spain; ^3^Pneumology Unit, University Clinic Hospital of Valencia, Valencia, Spain; ^4^Freshage Research Group, Department of Physiology, Faculty of Medicine, University of Valencia, CIBERFES, Fundación Investigación Hospital Clínico Universitario/INCLIVA, Valencia, Spain; ^5^CIBERDEM-Spanish Biomedical Research Center in Diabetes and Associated Metabolic Disorders, ISCIII, Madrid, Spain

**Keywords:** COVID-19, rale score, biomarkers, inflammation, SARS-CoV-2

## Abstract

**Background:**

Coronavirus disease 2019 (COVID-19) has placed enormous pressure on intensive care units (ICUs) and on healthcare systems in general. A deeper understanding of the pathophysiology of the most severe forms of COVID-19 would help guide the development of more effective interventions. Herein, we characterized the inflammatory state of patients with COVID-19 of varying degrees of severity to identify admission biomarkers for predicting COVID-19 worsening.

**Design:**

Admission blood samples were obtained from 78 patients with COVID-19. Radiographic assessment of lung edema (RALE) scoring was calculated by imaging. Platelet and leukocyte counts were measured by flow cytometry, and plasma levels of C-reactive protein were assessed by immunoturbidimetry, and interleukin (IL)-8/CXCL8, IL-10, tumor necrosis factor (TNF)-α, interferon (IFN)-γ, and *monocyte chemoattractant protein-1* (MCP-1/CCL2) levels by enzyme-linked immunosorbent assay (ELISA).

**Results:**

The RALE score correlated with several admission hemogram (platelets, neutrophils, and lymphocytes) and inflammatory (IL-8/CXCL8, MCP-1/CCL2, IL-10, and C-reactive protein) parameters. COVID-19 worsening, based on the need for oxygen (Δoxygen supply) during hospitalization, correlated negatively with admission lymphocyte counts but positively with neutrophil-to-lymphocyte ratio and with plasma levels of the inflammatory parameters correlating with RALE score.

**Conclusion:**

Our data indicate a correlation between the RALE score and Δoxygen supply and admission inflammatory status. The identification of a panel of biomarkers that reflect COVID severity might be useful to predict disease worsening during hospitalization and to guide clinical management of COVID-19-related complications. Finally, therapies targeting IL-8/CXCL8- or IL-10 activity may offer therapeutic approaches in COVID-19 treatment.

## Introduction

The coronavirus disease 2019 (COVID-19) pandemic caused by severe acute respiratory syndrome coronavirus-2 (SARS-CoV-2) infection has been unprecedented in terms of its global impact and the response to it (1). In the more vulnerable patients, SARS-CoV-2 infection can trigger a cascade of acute pathological events that ultimately necessitate mechanical ventilation and admission to the intensive care unit (ICU), and can lead to death (2). Indeed, while most of the reported hospital stays have been of short duration, the number of patients requiring ICU admission remains worryingly high. This has led to enormous pressure on ICUs and healthcare systems in general and has united researchers and clinicians in attempts to better understand the pathology of COVID-19 and the heterogeneity of the symptoms. Indeed, there is an unmet need to gain insights into clinical diagnosis criteria, such as laboratory biomarkers, to more accurately predict the most serious COVID-19 outcomes, which might reduce hospitalization rates and mortality.

Severe acute respiratory syndrome coronavirus-2 infection can trigger a cascade of biological events in patients, of which an acute inflammatory response is considered as a critical hallmark. This complex inflammatory response is due to an important innate and adaptive immune response that leads to an overexpression of pro-inflammatory cytokines, commonly known as *cytokine storm*, responsible for the severity of the COVID-19 immunopathology (3, 4). One of the most studied cytokines since the beginning of the pandemic, is interleukin (IL)-6 (3). Consistent results were obtained from several studies where plasma levels of IL-6 were found to be increased in COVID-19 patients being higher in severe cases (5–7). Of note, IL-6 *trans*-signaling is associated with pro-inflammatory responses (such as smooth muscle proliferation, endothelial dysfunction, and pro-inflammatory cytokine production and release (8, 9)) by the activation of the Janus kinases/signal transducer and activator of transcription protein 3 (JAK/STAT3) and phosphatidylinositol-3-kinase/protein kinase B or AKT (PI3K/AKT) signaling pathways (10). Indeed, JAK inhibitors have been suggested to control the cytokine storm in COVID-19 (11). While IL-6 plasma levels in COVID-19 patients have been widely studied during the pandemic and the results seemed to be consistent, some questions remain unanswered regarding the pattern of the cytokine profile in COVID-19. Nevertheless, the COVID-19-related *cytokine storm* is known to contribute to the mobilization and recruitment of macrophages and neutrophils which results in an acute respiratory distress syndrome (ARDS) (2, 12). Indeed, the high rate of neutrophilic infiltration in the pulmonary capillaries of COVID-19 patients has been reported to predict mortality (13). Additionally, this *cytokine storm* seems to contribute to T cell exhaustion which, along with T cell apoptosis directly induced by SARS-CoV-2 infection *via* ACE2, may be responsible for the lymphopenia observed in these patients (4, 14).

The evidence that the immune system might be insufficient against COVID-19 is compelling, which would explain the progressive organ damage seen in some patients and the significant associated lethality (15). The early detection of the heightened inflammatory state through monitoring circulating cytokines and other immune markers might provide an early therapeutic window in patients with COVID-19 who would otherwise evolve unfavorably. Described for the first time in 2018 by Warren et al. (16), the radiographic assessment of lung edema (RALE) score was first used as a non-invasive measurement of the pulmonary edema degree in 174 intubated patients with ARDS. The study showed that a low baseline RALE score was associated with better oxygenation, reflected by higher ratio of arterial oxygen partial pressure to fractional inspired oxygen (PaO_2_/FiO_2_), and longer survival in ARDS patients. In a recent *post-hoc* analysis (17), the correlation between RALE score and PaO_2_/FiO_2_ ratio was weak and the association between RALE score and mortality was not statistically significant. Although the ARDS is a consequence of SARS-CoV-2 infection (18) and its correlation with the RALE score is well-known; the relationship between RALE score and the inflammatory status has not yet been fully addressed.

Given the above, it seems clear that COVID-19 complications are closely linked to a proinflammatory state. In the present study, we aimed to characterize inflammation in patients with COVID-19 presenting with different degrees of severity. To do this, we stratified patients based on their admission RALE score, and on the oxygen supply applied at the time of admission and during the hospital stay. We then tested for relationships between RALE score and the inflammatory state in patients to identify admission biomarkers for predicting COVID-19 worsening, which may have the potential to guide therapeutic approaches to reduce hospitalization, deaths, and healthcare costs.

## Methods

### Human study population

The study complied with the principles outlined in the Declaration of Helsinki and was approved by the institutional ethics committee of the University Clinic Hospital of Valencia under the following code: 2020/349 (Valencia, Spain). In total, 78 patients with COVID-19 were included and recruited by the Pneumology Unit of the *University Clinic Hospital of Valencia* (Valencia, Spain) from April to December 2020. Inclusion criteria were as follows: (1) COVID-19 diagnosis confirmed by real-time reverse transcription polymerase chain reaction; (2) age ≥18 to ≤80 years; and (3) provision of written informed consent. Exclusion criteria were: (1) age <18 or >80 years; (2) days from symptoms onset >21; and (3) the presence of an active neoplastic disease.

Blood samples (EDTA) were drawn during the first few days of hospital admission (mean of 2.6 ± 0.4 days). Plasma was obtained by centrifugation method and stored at −80°C. All patients signed an informed consent. The demographic and clinical features of participants are shown in Table 1.

**TABLE 1:** Demographic and clinical features of participants.

	Group 0 (mild)	Group 1 (moderate)	Group 2 (severe)	*P*-value
Number per group (*n*)	27	44	7	–
Age (years)	54.26 ± 2.91	56.57 ± 2.11	62.14 ± 5.25	0.5691 (0 vs. 1) 0.4878 (0 vs. 2) 0.5691 (1 vs. 2)
Gender (male %)	51.85 %	79.55 %	85.71 %	–
Day of blood extraction after hospital admission	1.96 ± 0.35	2.89 ± 0.49	2.71 ± 1.11	0.4521 (0 vs. 1) 0.7783 (0 vs. 2) 0.8804 (1 vs. 2)
Number of days with symptoms at blood extraction	6.74 ± 0.77	6.93 ± 0.46	5.43 ± 0.78	0.8172 (0 vs. 1) 0.6212 (0 vs. 2) 0.6212 (1 vs. 2)

Data are presented as mean ± SEM.

The assessment of radiological involvement of each patient was calculated by adapting and simplifying the RALE score proposed by Warren et al. (16) and widely used (19): a score of 0–4 was assigned to each lung based on the extent of involvement by consolidation or ground-glass opacities. Also, patients were stratified into three groups according to their oxygen supply (oxygen_initial_) at the time of the blood extraction: without oxygen supply (group 0), nasal cannula/FiltaMask™ (group 1), or conventional oxygen therapy/non-invasive mechanical ventilation (group 2). We also registered the maximum oxygen therapy reached by each patient during the hospital stay (oxygen_maximum_). Disease progression (Δoxygen supply) was calculated for each patient as follows:


(1) $$\Delta \,\mathrm{oxygen}\,\mathrm{supply}={\mathrm{oxygen}}_{\text{maximum}}-{\mathrm{oxygen}}_{\text{initial}}$$

Patients were then restratified into the following additional groups: A (no changes in oxygen supply), B (moderate variation in oxygen supply), C (marked variation in oxygen supply).

### Blood counts of platelets and leukocyte subsets

The number of circulating platelets and leukocyte subsets (neutrophils, lymphocytes, monocytes, eosinophils, and basophils) was determined through a conventional hemogram using laser flow cytometry (Sysmex XN-9000, Sysmex, Kobe, Japan). Additionally, the neutrophil-to-lymphocyte ratio, a commonly used prognostic marker of COVID-19 progression (20, 21), was calculated.

### Determination of soluble inflammatory markers

Levels of human soluble interleukin (IL)-8/CXCL8, IL-10, *tumor necrosis factor* (TNF)-α, interferon (IFN)-γ, and *monocyte chemoattractant protein-1* (MCP-1/CCL2) were measured by enzyme-linked immunosorbent assay (ELISA; DuoSet^®^ or Quantikine^®^ ELISA Kits, R&D Systems, Abingdon, United Kingdom) in plasma from EDTA-treated whole blood. Plasma levels of C-reactive protein were measured by immunoturbidimetry (Beckman Coulter Au5800, Beckman Coulter, Pasadena, CA, United States). Results were expressed as mg or pg/ml of mediator in plasma.

### Statistical analysis

All results were analyzed using GraphPad Prism 6 (GraphPad Software, Inc., La Jolla, CA, United States). Values are expressed as individual data points or mean ± standard error of the mean (SEM) when appropriate. For comparisons of multiple groups, one-way analysis of variance followed by *post-hoc* Tukey’s analysis was used in data that passed both normality (Kolmogorov–Smirnov) and equal variance (Levene’s) tests; otherwise, the non-parametric Kruskal–Wallis test followed by Dunn’s *post-hoc* analysis was used. Data were considered statistically significant at *p* < 0.05. Correlations between experimental findings and RALE score or disease progression (Δoxygen supply) were calculated using the Pearson and Spearman correlation analysis procedures.

## Results

Of 99 patients initially recruited, 78 met the inclusion/exclusion criteria and were invited to participate. The distribution of patients according to RALE scores was as follows: *RALE score 0*, *n* = 3; *RALE score 1*, *n* = 5; *RALE score 2*, *n* = 12; *RALE score 3*, *n* = 15; *RALE score 4*, *n* = 15; *RALE score 5*, *n* = 13; *RALE score 6*, *n* = 7; *RALE score 7*, *n* = 4; and *RALE score 8*, *n* = 4. Patients were categorized into three groups based on their oxygen supply requirements at the moment of sample extraction: without oxygen supply (group 0, *n* = 27), nasal cannula/FiltaMask™ (group 1, *n* = 44), or conventional oxygen therapy/non-invasive mechanical ventilation (group 2, *n* = 7). The demographic and clinical features of the three groups are shown in Table 1. No significant differences between the three groups were found for age, day of blood extraction after hospital admission, or the number of days with symptoms at blood extraction.

### The radiographic assessment of lung edema score correlates positively with platelet and neutrophil counts and negatively with lymphocyte counts in patients with coronavirus disease 2019

**FIGURE 1:**
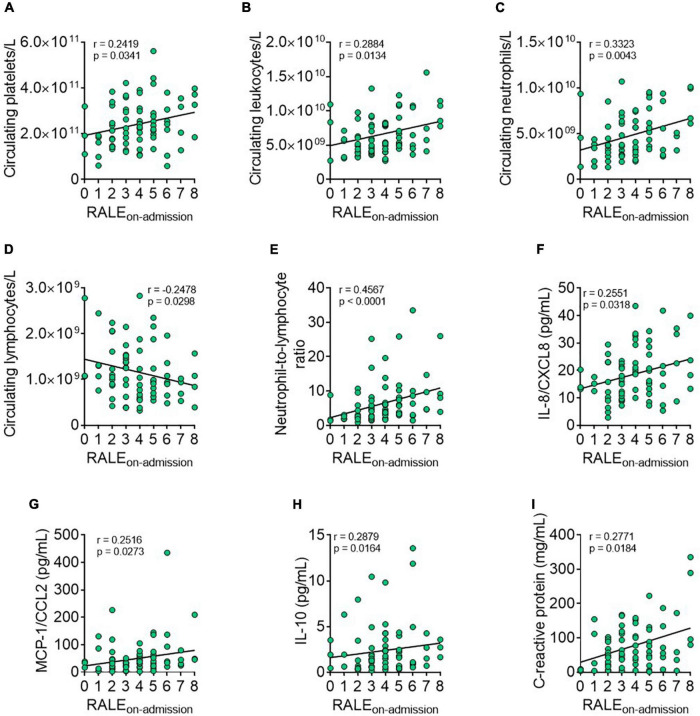
The radiographic assessment of lung edema (RALE) score correlates with several admission hemogram and inflammatory parameters in patients with coronavirus disease 2019 (COVID-19). Correlations between admission hemogram parameters [number of circulating platelets/L, **(A)** leukocytes/L, **(B)** neutrophils/L, **(C)** lymphocytes/L, **(D)** and neutrophil-to-lymphocyte ratio, **(E)**] or plasma levels of inflammatory mediators [IL-8/CXCL8, **(F)** *monocyte chemoattractant protein-1* (MCP-1/CCL2), **(G)** IL-10, **(H)**, and C-reactive protein, **(I)**] and the RALE score (calculated by radiographic imaging) were established using the Pearson/Spearman correlation analysis procedures. Values are expressed as individual data points (*n* = 78 patients). Data were considered statistically significant at *p* < 0.05.

We investigated the relationship between blood counts of circulating platelets and leukocyte subsets determined by flow cytometry and the RALE score in patients at admission, finding a positive correlation between the RALE score and circulating platelets (Figure 1A). A comparable result was found for the RALE score and circulating leukocytes (Figure 1B), which appeared to be due to the neutrophil population (Figure 1C) as the RALE score and lymphocyte counts showed a negative correlation (Figure 1D). Consequently, the RALE score positively correlated with neutrophil-to-lymphocyte ratio (Figure 1E), a common prognostic marker of COVID-19 progression (20, 21). No correlations were found between the RALE score and other leukocyte subpopulations (eosinophils and basophils, data not shown). We next measured the levels of six relevant soluble inflammatory mediators in the plasma of patients at hospital admission. Results showed that the admission plasma level of the neutrophil chemotactic cytokine IL-8/CXCL8, the monocyte chemoattractant cytokine MCP-1/CCL2, the anti-inflammatory cytokine IL-10, and the acute-phase inflammatory marker C-reactive protein correlated positively with the RALE score (Figures 1F–I). No correlations were found between the RALE score and TNFα or IFNγ plasma levels (data not shown).

### Oxygen supply requirement and the radiographic assessment of lung edema score are both indicators of coronavirus disease 2019 severity

**FIGURE 2:**
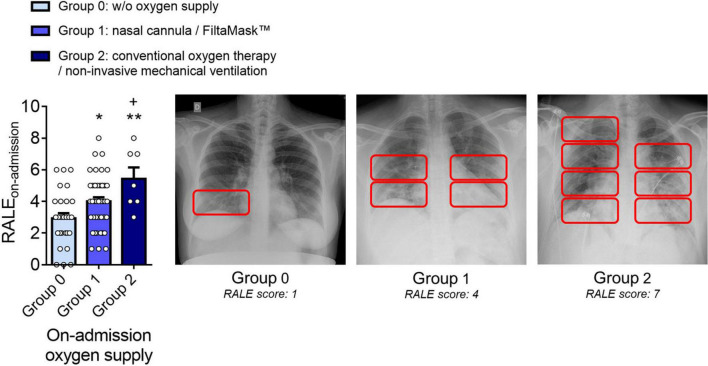
The RALE score correlates with the oxygen supply administrated, validating oxygen supply as an indicator of COVID-19 severity. Distribution of the RALE score according to admission oxygen supply groups: group 0 (mild COVID-19, without oxygen supply, *n* = 27), group 1 (moderate COVID-19, nasal cannula/FiltaMask™, *n* = 44) and group 2 (severe COVID-19, conventional oxygen therapy/non-invasive mechanical ventilation, *n* = 7). Representative radiographic images with the respective RALE scoring are shown. Values are expressed as mean ± SEM. *p < 0.05 or **p < 0.01 relative to group 0, and +*p* < 0.05 relative to group 1.

We next investigated the distribution of the RALE score in patients according to the admission oxygen supply group, finding that it was significantly higher in group 2 than in groups 0 and 1, and was significantly higher in group 1 than in group 0 (Figure 2). Representative radiographic images with the respective RALE scoring among groups 0, 1, and 2 are shown in Figure 2. We classified groups 0, 1, and 2 as mild, moderate, and severe COVID-19 groups, respectively.

### Coronavirus disease 2019 worsening during hospital stay (Δoxygen supply) correlates negatively with admission lymphocyte counts but positively with admission neutrophil-to-lymphocyte ratio and with plasma levels IL-8/CXCL8, *monocyte chemoattractant protein-1*/CCL2, IL-10, and c-reactive protein

**FIGURE 3:**
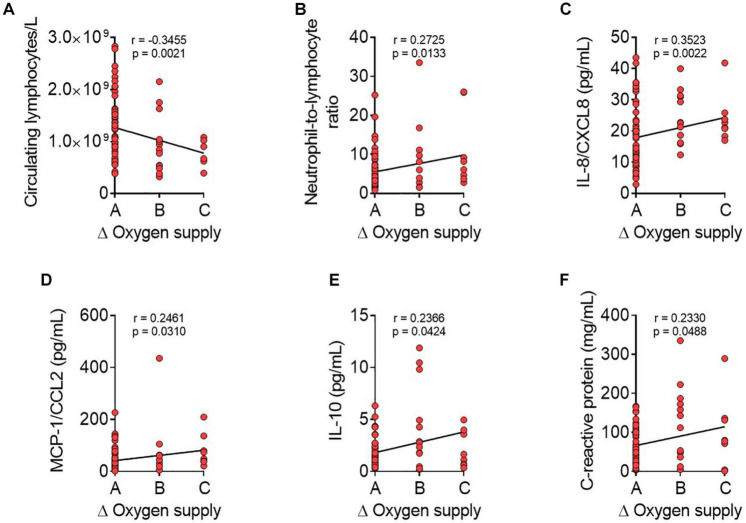
Coronavirus disease 2019 worsening during hospitalization is correlates negatively with admission lymphocyte counts but positively with admission neutrophil-to-lymphocyte ratio, IL-8/CXCL8, MCP-1/CCL2, IL-10, and C-reactive protein plasma levels. Correlations between admission hemogram parameters [number of circulating lymphocytes/L, **(A)** and neutrophil-to-lymphocyte ratio, **(B)**] or plasma levels of inflammatory mediators [IL-8/CXCL8, **(C)** MCP-1/CCL2, **(D)** IL-10, **(E)**, and C-reactive protein, **(F)**] and disease progression (Δoxygen supply) were calculated using Pearson/Spearman correlation analysis procedures. Disease progression was calculated for each participant by: maximum oxygen therapy during the hospital stay–oxygen therapy at hospital-admission blood extraction (Δoxygen supply). Group A = no changes in oxygen supply; group B = moderate variation in oxygen supply; group C = marked variation in oxygen supply. Values are expressed as individual data points (*n* = 78 patients). Data were considered statistically significant at *p* < 0.05.

To search for novel predictors of COVID-19 severity, we evaluated the disease progression (Δoxygen supply) for each patient based on the initial and the maximum oxygen supply administered during the hospital stay. This analysis produced the following three additional groups: group A (no changes in oxygen supply, *n* = 56), group B (moderate variation in oxygen supply, *n* = 13), and group C (marked variation in oxygen supply, *n* = 9). We then re-analyzed the blood data at admission using this new stratification. Notably, whereas admission platelet, total leukocyte, neutrophil, monocyte, eosinophil, or basophil counts showed no correlation with disease progression (data not shown), admission lymphocyte counts negatively correlated with COVID-19 worsening (Figure 3A). Contrastingly, we found a positive correlation between the admission neutrophil-to-lymphocyte ratio and disease worsening (Figure 3B). Finally, COVID-19 worsening during hospitalization is also positively correlated with admission plasma levels of IL-8/CXCL8 (Figure 3C), MCP-1/CCL2 (Figure 3D), IL-10 (Figure 3E), and C-reactive protein (Figure 3F).

## Discussion

The RALE score has proven to be useful in the diagnosis of acute respiratory distress syndrome (17), a complication of SARS-Cov-2 infection (18). However, little is known about the potential relationship between the RALE score and the initial inflammatory status in these patients. In the present study, we found that the admission RALE score positively correlated with several hemogram and inflammatory parameters in patients with COVID-19, which has not been previously addressed. Although a link between platelet counts and COVID-19 severity has been broadly described, these findings remain controversial. Indeed, while several reports indicated that platelet counts were lower in patients with severe COVID-19 than in peers with mild disease (22–24), the opposite has also been reported in patients with compromised lung function (25). We found that the admission platelet counts positively correlated with the RALE score, indicating that patients with a greater degree of compromised lung function have elevated platelet counts. It should be highlighted, however, that this determination was done at the time of hospital admission and a follow-up analysis would be required to fully understand the evolution of platelet counts along the disease progression and the hospital stay.

We also show that the RALE score positively correlates with admission leukocyte counts. This appears to be based on the neutrophil population, as no other positive correlation was detected with other leukocyte subsets. Indeed, a negative correlation between the RALE score and admission lymphocyte counts was observed. Consequently, the RALE score also positively correlated with the neutrophil-to-lymphocyte ratio, a common prognostic marker of COVID-19 progression (20, 21). Overall, these results suggest that COVID-19 severity is accompanied by an elevated neutrophil count and a low lymphocyte count, which is in agreement with recently published data (24).

In line with these observations, the admission levels of IL-8/CXCL8, a neutrophil chemoattractant involved in inflammatory processes (26), positively correlated with the RALE score, which might explain the neutrophilia observed in patients with compromised lung function. Indeed, elevated levels of IL-8/CXCL8 have also been documented in patients with severe COVID-19, both in plasma (27) and in lung samples (12). IL-8/CXCL8 can exert beneficial effects in pathogen elimination, but it can also contribute to tissue injury and fibrosis (26). Another chemokine found to positively correlate with the RALE score was MCP-1/CCL2, which has chemoattractant properties for CCR2-expressing monocytes and macrophages (28), whereas no correlation was found between the RALE score and admission monocyte counts, MCP-1/CCL2 is associated with monocyte-derived macrophage infiltration in the lungs of patients with COVID-19 and is increased in those patients with acute respiratory distress syndrome in which it appears to play a key role in pulmonary fibrosis development (29).

Unexpectedly, we found that admission IL-10 plasma levels also positively correlated with the RALE score despite the fact that it is a well-known anti-inflammatory cytokine. Although this observation appears to be contradictory, high levels of this cytokine have been detected in patients with COVID-19, and were greater in critical patients than in moderate/severe groups (5). IL-10 can neutralize the activity of macrophages and Th1 and natural-killer lymphocytes, and can counter the production and release of relevant proinflammatory mediators, such as IL-8/CXCL8, TNFα, IFNγ, and MCP-1/CCL2. We found that the circulating levels of these cytokines/chemokines were either unaffected or were even elevated in severe disease. Indeed, circulating T-regulatory cells, a typical IL-10-producing cell, have been found to be largely increased in abundance in severe COVID-19 despite the concomitant lymphopenia (14). Accordingly, in the scenario of COVID-19, IL-10 might worsen the disease outcome rather than produce a benefit. Finally, the admission levels of the acute-phase inflammatory marker C-reactive protein also positively correlated with the RALE score. Although this observation has not been previously described, a recent study reported decreased C-reactive protein levels in patients with COVID-19 and a low RALE score (30), in agreement with our findings.

We analyzed the differences in the RALE score among groups based on oxygen supply to validate the patient distribution for oxygen supply as an indicator of COVID-19 severity. We established three additional groups–A, B, and C (no changes, moderate variation, and marked variation in oxygen supply, respectively)–to look for novel COVID-19 worsening predictors. From the Pearson/Spearman correlation analyses, we conclude that low admission lymphocyte count and high admission neutrophil-to-lymphocyte ratio are predictors of COVID-19 worsening. Additionally, positive correlations between admission IL-8/CXCL8, MCP-1/CCL2, IL-10, and C-reactive protein plasma levels and Δoxygen supply during hospitalization could predict COVID-19 worsening, and some studies partly corroborate these findings (31–34).

Taken together, our findings suggest that admission values of lymphocyte counts, neutrophil-to-lymphocyte ratio and the levels of some inflammatory markers (IL8/CXCL8, MCP-1/CCL2, IL-10, and C-reactive protein) may be useful as COVID-19 outcome predictors during hospitalization. In addition, our results might yield new therapeutic strategies. As IL-8/CXCL8 circulating levels are elevated in COVID-19 patients with high lung function impairment (high admission RALE score) along with neutrophil counts, IL-8/CXCL8 neutralization might exert beneficial effects in pulmonary complications of the disease. In this context, a phase-2 clinical trial with a monoclonal antibody against IL-8/CXCL8 (HuMax IL8–BMS-986253) is currently ongoing in the United States (35). By contrast, anti-IL-10 therapy has not yet been attempted as far as we know, likely due to its unclear role in this disease.

In conclusion, we provide the first evidence that the RALE score and Δoxygen supply correlate with the admission inflammatory status of patients with COVID-19, which can predict COVID-19 worsening during hospitalization. These data might inform clinical approaches to reduce COVID-19-related complications. Finally, our data may have an impact on the use of new therapeutic tools targeting IL-8/CXCL8- and/or IL-10 activity.

It is noteworthy to point out that this work has some limitations. According to the last update of the *European Centre for Disease Prevention and Control* (ECDC) on February 24th 2022, more than 420 million cases of COVID-19 have been reported worldwide since the beginning of the pandemic (36). However, only 78 patients from a single medical center were included in this study, which can represent a limitation for the extrapolation of the results and conclusions obtained. Moreover, although significant correlations were obtained from the present study, the correlation coefficients only indicate weak-to-moderate correlations (*R*-values from 0.2330 to 0.4567) (37). Both limitations could be solved by increasing the sample size from an international multicenter study. Nevertheless, the results and conclusions herein presented might have an impact on the clinical routine or future research direction.

## Data availability statement

The raw data supporting the conclusions of this article will be made available by the authors, without undue reservation.

## Ethics statement

The studies involving human participants were reviewed and approved by the Ethic Committee of Clinical Research, University Clinic Hospital of Valencia, Spain. The patients/participants provided their written informed consent to participate in this study.

## Author contributions

M-JS, JS-C, and JV designed research and had primary responsibility for final content. PM, LF-P, and AC performed research. PM and LF-P analyzed and interpreted the data. PM, LF-P, JV, J-SC, and M-JS drafted the manuscript. AC and M-CG-C revised it critically for important intellectual content. All authors have read and approved the final version of the manuscript.

## References

[1] Josephson A Kilic T Michler JD. Socioeconomic impacts of COVID-19 in low-income countries. *Nat Hum Behav.* (2021) 5:557–65. 10.1038/s41562-021-01096-7 33785897

[2] Attaway AH Scheraga RG Bhimraj A Biehl M Hatipoğlu U. Severe covid-19 pneumonia: Pathogenesis and clinical management. *BMJ.* (2021) 372:n436. 10.1136/bmj.n436 33692022

[3] Tang Y Liu J Zhang D Xu Z Ji J Wen C. Cytokine storm in COVID-19: The current evidence and treatment strategies. *Front Immunol.* (2020) 11:1708. 10.3389/fimmu.2020.01708 32754163 PMC7365923

[4] Yang L Xie X Tu Z Fu J Xu D Zhou Y. The signal pathways and treatment of cytokine storm in COVID-19. *Signal Transduct Target Ther.* (2021) 6:255. 10.1038/s41392-021-00679-0 34234112 PMC8261820

[5] Han H Ma Q Li C Liu R Zhao L Wang W Profiling serum cytokines in COVID-19 patients reveals IL-6 and IL-10 are disease severity predictors. *Emerg Microbes Infect.* (2020) 9:1123–30. 10.1080/22221751.2020.1770129 32475230 PMC7473317

[6] Chen G Wu D Guo W Cao Y Huang D Wang H Clinical and immunological features of severe and moderate coronavirus disease 2019. *J Clin Invest.* (2020) 130:2620–9. 10.1172/jci137244 32217835 PMC7190990

[7] Diao B Wang C Tan Y Chen X Liu Y Ning L Reduction and functional exhaustion of T cells in patients with coronavirus disease 2019 (COVID-19). *Front Immunol.* (2020) 11:827. 10.3389/fimmu.2020.00827 32425950 PMC7205903

[8] Luo JY Fu D Wu YQ Gao Y. Inhibition of the JAK2/STAT3/SOSC1 signaling pathway improves secretion function of vascular endothelial cells in a rat model of pregnancy-induced hypertension. *Cell Physiol Biochem.* (2016) 40:527–37. 10.1159/000452566 27889763

[9] Kang S Tanaka T Inoue H Ono C Hashimoto S Kioi Y IL-6 trans-signaling induces plasminogen activator inhibitor-1 from vascular endothelial cells in cytokine release syndrome. *Proc Natl Acad Sci USA.* (2020) 117:22351–6. 10.1073/pnas.2010229117 32826331 PMC7486751

[10] Zegeye MM Lindkvist M Fälker K Kumawat AK Paramel G Grenegård M Activation of the JAK/STAT3 and PI3K/AKT pathways are crucial for IL-6 trans-signaling-mediated pro-inflammatory response in human vascular endothelial cells. *Cell Commun Signal.* (2018) 16:55. 10.1186/s12964-018-0268-4 30185178 PMC6125866

[11] Luo W Li YX Jiang LJ Chen Q Wang T Ye DW. Targeting JAK-STAT signaling to control cytokine release syndrome in COVID-19. *Trends Pharmacol Sci.* (2020) 41:531–43. 10.1016/j.tips.2020.06.007 32580895 PMC7298494

[12] Azevedo MLV Zanchettin AC Vaz de Paula CB Motta Júnior JdS Malaquias MAS Raboni SM Lung neutrophilic recruitment and IL-8/IL-17A tissue expression in COVID-19. *Front Immunol.* (2021) 12:656350. 10.3389/fimmu.2021.656350 33868301 PMC8044579

[13] Laforge M Elbim C Frère C Hémadi M Massaad C Nuss P Tissue damage from neutrophil-induced oxidative stress in COVID-19. *Nat Rev Immunol.* (2020) 20:515–6. 10.1038/s41577-020-0407-1 32728221 PMC7388427

[14] Neumann J Prezzemolo T Vanderbeke L Roca CP Gerbaux M Janssens S Increased IL-10-producing regulatory T cells are characteristic of severe cases of COVID-19. *Clin Transl Immunol.* (2020) 9:e1204. 10.1002/cti2.1204 33209300 PMC7662088

[15] Schönrich G Raftery MJ Samstag Y. Devilishly radical NETwork in COVID-19: Oxidative stress, neutrophil extracellular traps (NETs), and T cell suppression. *Adv Biol Regul.* (2020) 77:100741. 10.1016/j.jbior.2020.100741 32773102 PMC7334659

[16] Warren MA Zhao Z Koyama T Bastarache JA Shaver CM Semler MW Severity scoring of lung oedema on the chest radiograph is associated with clinical outcomes in ARDS. *Thorax.* (2018) 73:840–6. 10.1136/thoraxjnl-2017-211280 29903755 PMC6410734

[17] Zimatore C Pisani L Lippolis V Warren MA Calfee CS Ware LB Accuracy of the radiographic assessment of lung edema score for the diagnosis of ARDS. *Front Physiol.* (2021) 12:672823. 10.3389/fphys.2021.672823 34122143 PMC8188799

[18] Fan E Beitler JR Brochard L Calfee CS Ferguson ND Slutsky AS COVID-19-associated acute respiratory distress syndrome: Is a different approach to management warranted? *Lancet Respir Med.* (2020) 8:816–21. 10.1016/s2213-2600(20)30304-0 32645311 PMC7338016

[19] Wong HYF Lam HYS Fong AH-T Leung ST Chin TW-Y Lo CSY Frequency and distribution of chest radiographic findings in patients positive for COVID-19. *Radiology.* (2020) 296:E72–8. 10.1148/radiol.2020201160 32216717 PMC7233401

[20] Jimeno S Ventura PS Castellano JM García-Adasme SI Miranda M Touza P Prognostic implications of neutrophil-lymphocyte ratio in COVID-19. *Eur J Clin Invest.* (2021) 51:e13404. 10.1111/eci.13404 32918295

[21] Ciccullo A Borghetti A Zileri Dal Verme L Tosoni A Lombardi F Garcovich M Neutrophil-to-lymphocyte ratio and clinical outcome in COVID-19: A report from the Italian front line. *Int J Antimicrob Agents.* (2020) 56:106017. 10.1016/j.ijantimicag.2020.106017 32437920 PMC7211594

[22] Huang J Gao J Zhu W Feng R Liu Q Chen X Indicators and prediction models for the severity of Covid-19. *Int J Clin Pract.* (2021) 75:e14571. 10.1111/ijcp.14571 34170611 PMC8420422

[23] Szklanna PB Altaie H Comer SP Cullivan S Kelliher S Weiss L Routine hematological parameters may be predictors of COVID-19 severity. *Front Med (Lausanne).* (2021) 8:682843. 10.3389/fmed.2021.682843 34336889 PMC8322583

[24] Az A Sogut O Akdemir T Ergenc H Dogan Y Cakirca M. Impacts of demographic and clinical characteristics on disease severity and mortality in patients with confirmed COVID-19. *Int J Gen Med.* (2021) 14:2989–3000. 10.2147/ijgm.S317350 34234528 PMC8254610

[25] Lanza E Mancuso ME Messana G Ferrazzi P Lisi C Di Micco P Compromised lung volume and hemostatic abnormalities in COVID-19 pneumonia: Results from an observational study on 510 consecutive patients. *J Clin Med.* (2021) 10:2894. 10.3390/jcm10132894 34209720 PMC8268714

[26] Russo RC Garcia CC Teixeira MM Amaral FA. The CXCL8/IL-8 chemokine family and its receptors in inflammatory diseases. *Expert Rev Clin Immunol.* (2014) 10:593–619. 10.1586/1744666x.2014.894886 24678812

[27] Alosaimi B Mubarak A Hamed ME Almutairi AZ Alrashed AA AlJuryyan A Complement anaphylatoxins and inflammatory cytokines as prognostic markers for COVID-19 severity and in-hospital mortality. *Front Immunol.* (2021) 12:668725. 10.3389/fimmu.2021.668725 34276659 PMC8281279

[28] Deshmane SL Kremlev S Amini S Sawaya BE. Monocyte chemoattractant protein-1 (MCP-1): An overview. *J Interferon Cytokine Res.* (2009) 29:313–26. 10.1089/jir.2008.0027 19441883 PMC2755091

[29] Wendisch D Dietrich O Mari T von Stillfried S Ibarra IL Mittermaier M SARS-CoV-2 infection triggers profibrotic macrophage responses and lung fibrosis. *Cell.* (2021) 184:6243–6261.e27. 10.1016/j.cell.2021.11.033 34914922 PMC8626230

[30] Sigman SA Mokmeli S Vetrici MA. Adjunct low level laser therapy (LLLT) in a morbidly obese patient with severe COVID-19 pneumonia: A case report. *Can J Respir Ther.* (2020) 56:52–6. 10.29390/cjrt-2020-022 33043132 PMC7521601

[31] Gallo Marin B Aghagoli G Lavine K Yang L Siff EJ Chiang SS Predictors of COVID-19 severity: A literature review. *Rev Med Virol.* (2021) 31:1–10. 10.1002/rmv.2146 32845042 PMC7855377

[32] Li L Li J Gao M Fan H Wang Y Xu X Interleukin-8 as a biomarker for disease prognosis of coronavirus disease-2019 patients. *Front Immunol.* (2020) 11:602395. 10.3389/fimmu.2020.602395 33488599 PMC7820901

[33] Chen Y Wang J Liu C Su L Zhang D Fan J IP-10 and MCP-1 as biomarkers associated with disease severity of COVID-19. *Mol Med.* (2020) 26:97. 10.1186/s10020-020-00230-x 33121429 PMC7594996

[34] Dhar SK Damodar S Gujar S Das M. IL-6 and IL-10 as predictors of disease severity in COVID-19 patients: Results from meta-analysis and regression. *Heliyon.* (2021) 7:e06155. 10.1016/j.heliyon.2021.e06155 33553782 PMC7846230

[35] Patel S Saxena B Mehta P. Recent updates in the clinical trials of therapeutic monoclonal antibodies targeting cytokine storm for the management of COVID-19. *Heliyon.* (2021) 7:e06158. 10.1016/j.heliyon.2021.e06158 33553708 PMC7846241

[36] ECDC. *European centre for disease prevention and control: COVID-19 situation update worldwide 2022.* Frösunda: ECDC (2022).

[37] Schober P Boer C Schwarte LA. Correlation coefficients: Appropriate use and interpretation. *Anesth Analg.* (2018) 126:1763–8. 10.1213/ane.0000000000002864 29481436

